# Online Moisture Measurement of Bio Fuel at a Paper Mill Employing a Microwave Resonator †

**DOI:** 10.3390/s18113844

**Published:** 2018-11-09

**Authors:** Martta-Kaisa Olkkonen

**Affiliations:** Department of Electrical Engineering and Automation, Aalto University, Maarintie 8, 02150 Espoo, Finland; martta-kaisa.olkkonen@alumni.aalto.fi

**Keywords:** bio fuel, microwave resonator, moisture measurement, paper mill

## Abstract

This paper investigates online moisture measurement of biofuel employing a strip line cavity resonator at approximately 366 MHz, attached above and below the conveyor belt. An existing sensor design is modified for the factory assembly, and the correct operation has been tested prior to this paper with a small number of measurement points and collected reference samples (*n* = 67). The purpose is now to concentrate on the accuracy of the measurement and increase the number of measurement points (*n* = 367). The measurements were made in 5 different lots, and the thickness and moisture properties of the biomaterial mat were varied between minimum and maximum levels by adjusting the settings of the belt filter press that presses pulp slush into a mat. In order to further reduce inaccuracy, at the maximum one standard deviation was allowed from the average height of the equivalent water layer for each dataset, and consequently the number of samples was reduced to 235. A linear fit and a parabola fit were determined for thickness of the equivalent water layer vs. the relative resonant frequency shift: R^2^ = 0.82 and R^2^ = 0.78.

## 1. Introduction

### 1.1. Microwave Moisture Measurement of Biomaterials

Moisture content monitoring is a method for evaluating an industrial process; this paper investigates online moisture measurement of biofuel conducted in a paper factory. Moisture measurements of biomaterials such as paper are discussed in [1,2]. The biomaterial is slush of the paper process and it is pressed into a mat in a belt filter press used in biological treatment plants for processing pulp slush. It is characterized by a porous, inhomogeneous structure and also a high ion content due to impurities, mainly salts. For example [3] studied quality assessment of the paper manufacturing process. On-line moisture measurements are challenging, because the material under test moves constantly as it passes the sensor on a conveyor belt or in a pipe. Ref. [4] reports moisture content monitoring in paper and veneer manufacturing processes employing similar kinds of resonator sensors as in the current research. A resonator sensor was patented (2012) for measuring the moisture content of a mat in the wire end of a paper machine [5]. However, in our present application, an extra variable compared to plywood is thickness, because the belt filter press is not designed to adjust the thickness of the biomaterial accurately, as typically it is not a relevant parameter*.* This feasibility study on moisture evaluation of biofuel is especially challenging because it is conducted as an on-line measurement and the material is rather inhomogeneous. The mineral content of biomaterials can vary highly and finding a correlation between an electrical parameter and the water content requires a proper statistical analysis.

Analysis of biofuel drying was carried out in [6] for wood-based masses and in [7] a case study was carried out for tea leaves. Typical biofuels are bark, forest residue, sawdust and crushed construction wood [6]. The microwave moisture measurement methods are well-established for wood-base biofuels, since the measurement instrumentation of for example timber is already found in the literature [8]. The moisture content generally varies from 45–55% (wet-basis) in biofuel but this causes combustion problems in power plants [9]. The price of biofuel is determined by the moisture content and, in addition, water is not a part of the combustion process; instead it only absorbs heat energy during the burning. Typical methods for measuring the moisture content are either fuel flow online or fuel bulk in a large container [10].

One traditional and destructive moisture evaluation method is oven-drying, where a sample is taken from the conveyor belt and dried in the oven; for wood the standard temperature is 102–105 degrees Celsius. The percentage moisture content is calculated on a wet basis as a ratio of mass of water to the total mass of moist material (gravimetric)

(1) $$M=\frac{m_{w}}{m_{w}+m_{d}}\times 100\%,$$

where *m_w_* is the mass of water and *m_d_* is the mass of dry material.

### 1.2. Definition of Electric Parameters

The complex permittivity of an isotropic material is

(2) $$\varepsilon =\varepsilon_{r}\varepsilon_{0}=\varepsilon_{0}(\varepsilon_{r}^{\prime }-j\varepsilon_{r}^{\prime \prime }),$$

where $\varepsilon_{r}^{\prime }$ is the real part and $\varepsilon_{r}^{\prime \prime }$ is the imaginary part of the complex relative permittivity and the permittivity of vacuum is $\varepsilon_{0}$ ≈ 8.854 × 10^−12^ F/m.

Let us define the parameters that are used for deriving the material properties from measurement with a strip line cavity resonator sensor. The perturbation equations [11] (pp. 141–145) assume that the electric field is approximately unchanged when the change in permittivity of material is small. The electric field needs to be constant inside the sample that is inserted in the resonator. Even mode occurs when the electric field is tangential to the sample. The following approximation for the relative change of frequency (3) applies for the even wave mode [11]:

(3) $$\frac{\Delta f_{r}}{f_{r}}\approx -\frac{\varepsilon_{r}^{\prime }-1}{2}S,$$

when the relative magnetic permeability is $\mu_{r}\approx 1$ and *S* is the filling factor of the resonator and it depends on the dimensions of the sample. Equation (3) is essentially a linear approximation of the frequency dependency on the real part of relative permittivity, since it assumes a low loss case, so that $\varepsilon_{r}^{\prime }$ *>>* $\varepsilon_{r}^{\prime \prime }$.

The moisture measurement is conducted as a single-parameter measurement according to (3) that is not dependent on $\varepsilon_{r}^{\prime \prime }$. The real part of permittivity is simply related to the resonant frequency shift caused by inserting dielectric material in the resonator cavity. As a further approximation, the only variable is limited to the changing water content in the material. Thus, we aim to find a statistical correlation directly between the moisture content and the resonant frequency shift of the resonator.

### 1.3. Objective of Research

The pulp slush is first treated by adding polymers, which make the slush lose water more easily. The moisture measurement is conducted online, but the material is also conveyed to a combustion chamber before burning. Therefore, an interesting result of this research is not the moisture content at different points of the mat but, instead, the total amount of water in the container. In other words, the objective of this work is not to find wet patches on the mat, but instead to estimate the average moisture content of the material that passes the sensor. Based on the moisture measurement, it is possible to estimate the amount of water in the container, but many samples need to be recorded so that the measurement would be representative of the bulk [9]. Since the microwave moisture measurement represents a volumetric method, the process can be monitored during a specified time and can estimate the amount of water of the bulk.

The requirement specifications for the project are as follows: for the biofuel plant, the goal would be to find an optimal level for the dehumidification of the biomaterial prior to burning. The primary objective of burning the biomaterial is to minimize its volume, which reduces the disposal costs. The heat energy is significant for the plant only if the dry material content in the biomaterial mat is sufficient. The dry material content, which is achieved by mechanical pressing, is usually between 20–40%, an optimum being 50% for the burning according to the biofuel plant. Thus, the moisture content needs to be monitored in order to find an optimum balance where the costs of burning the moist material are lower than the possible savings in energy. If the accuracy of the moisture measurement is sufficient, the paper factory can decide whether the biomaterial is burnt for energy or disposed of. Also, the process parameters of the belt filter press could be more easily adjusted according to the moisture readings. The main objective of this first experiment was to investigate, whether the chosen sensor type could be used for monitoring the moisture content of the biomaterial that differs from paper or wood. Even after pressing, the moisture content of the biomaterial can reach up to 60%, whereas for paper it stays usually under 10%. The biggest difference compared to paper is a large ionic impurity content, mainly salts, which cause conductive losses.

## 2. Materials and Methods

Online moisture measurement requires a non-contacting transmission-type resonator; Material flows through the sensor and the so-called transmission scattering parameters are measured and analyzed. Due to inhomogeneity of the material, a relatively large footprint of the order of 300 cm^2^ is chosen in order to obtain an average result of the footprint area. This leads to choosing the frequency range of the measurement (~400 MHz) to be rather low. The chosen sensor type for this first feasibility study is an existing strip line cavity resonator design [12], which was modified for the factory assembly. This kind of transmission measurement averages a volume that can be called an equivalent volume element. This volume element is determined by the footprint area of the measurement and the thickness of the mat. In online sensing the speed of the measurement should be optimal so that the device does not move more than the area of the footprint during the recording of one measurement point. The permittivity value becomes a “sliding average” if the speed of the measurement device is too high compared to the measurement speed. However, this can be allowed according to the requirement specification of finding the volumetric water content of the material that is burnt.

### 2.1. Modification of the Existing Sensor Design and Simulations

The chosen sensor is a λ/4 strip line cavity resonator, and the field lines appear as in a strip transmission line. It was patented in [12] and in commercial use for measuring the moisture content of plywood [13]. A similar resonator was used for measurement of a paper web in [14]. The one-conductor strip line resonator supports only the odd mode and the two-conductor λ/4 strip line resonator supports only the even mode [14]. The odd and even modes are partly sensitive to different parameters of the material. For this resonator, the relative resonant frequency shift is derived from the perturbation theory [11].

**Figure 1:**
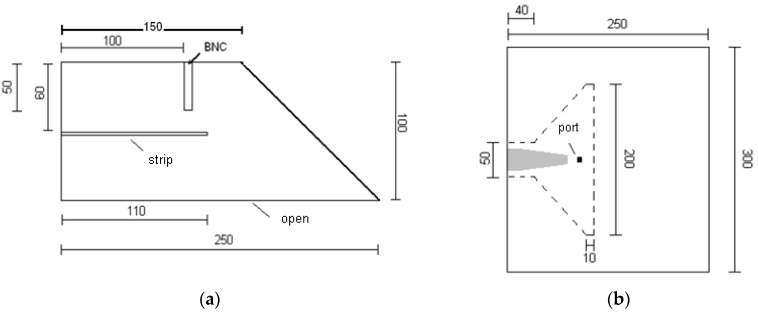
(**a**) Side view of a resonator half; (**b**) top view of a resonator half. Dimensions are in millimeters.

The dimensions of the resonator halves are 250 mm × 300 mm × 100 mm and the strip is placed at 60 mm distance from the bottom of both resonator halves. It was not possible to assemble the existing rectangular resonator to the belt filter press and, therefore, as a modification to the original design, one edge of both resonator halves was made slanted. The dimensions of a resonator half in millimeters are shown from the side in Figure 1a and from the top in Figure 1b. 

Operation of the resonator was simulated and the distance between the resonator halves was also set to 60 mm. According to the simulations, the slant edge had negligible effect on the resonator operation because the resonant frequency was 359.5 MHz for the rectangular resonator and 357.5 MHz with slant edges, respectively.

**Figure 2:**
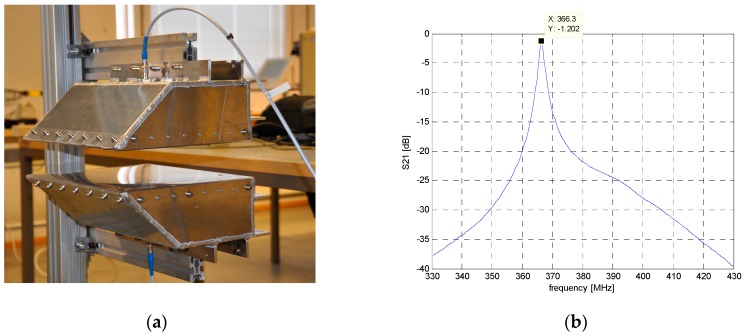
(**a**) Resonator in the laboratory; (**b**) measured resonance curve of the empty resonator.

Preliminary tests with the modified sensor design were carried out in [15,16]. Figure 2a presents the resonator construction during laboratory tests, where the resonant frequency was measured without the material under test (MUT) and it was *f_r_*_0_ = 366.3 MHz. The vector network analyzer employed in the laboratory and also in the factory experiments was HP8753D. Figure 2b shows the resonant curve of the empty resonator; the insertion loss is 1.2 dB at the resonant frequency. The resonant frequency is determined accurately only from a sharp resonance. Insertion loss varied during the measurement campaign of the biofuel between 26 and 24 dB of those samples that showed a sharp detectable resonance; other samples were not included in the analysis.

### 2.2. Online Moisture Measurement of Biofuel

**Figure 3:**
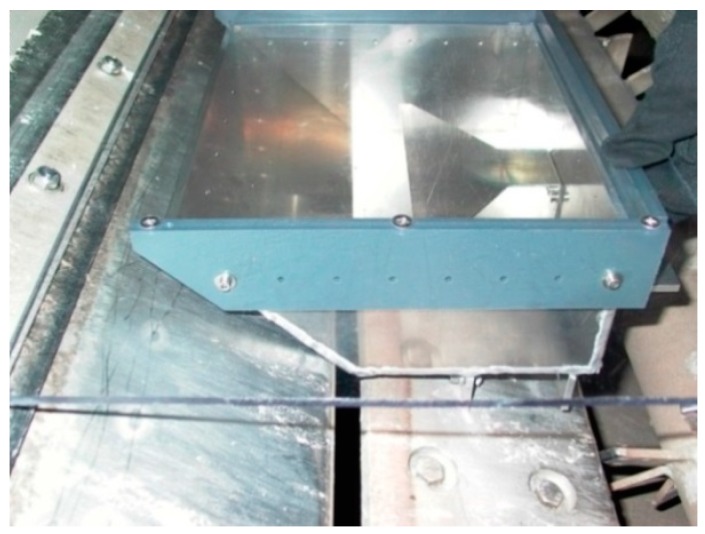
Resonator half assembled to the belt filter press.

The sensor was assembled to the end part of a belt filter press, where the mat falls to a ripper, cutting the mat into small pieces and after that the material is moved to a combustion chamber. Figure 3 shows the lower resonator half and the center conductor or “strip”. The strips of the resonator were made wider (200 mm) to enlarge the measurement footprint. A Plexiglas protects the cavity and directs the mat to flow evenly through the resonator halves. 

**Figure 4:**
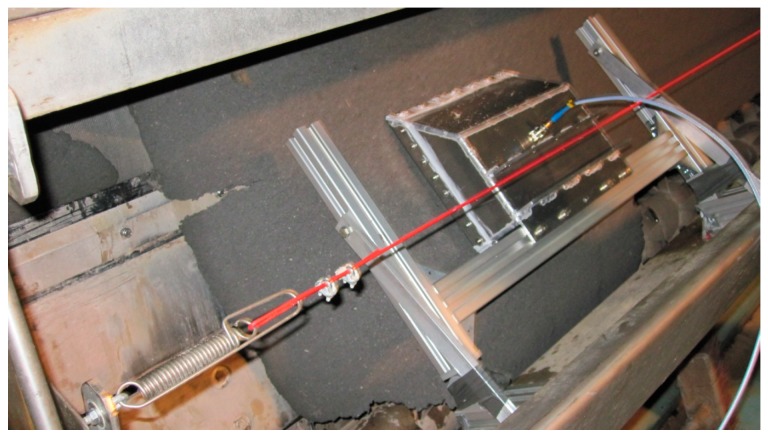
Biomaterial mat moving through the resonator halves during the factory experiments.

Figure 4 illustrates how the biomaterial mat flows through the resonator halves in the online measurement. The sensor was placed in the middle of the mat and the measurement was made along one longitudinal line. The uncertainty of the relative resonant frequency shift was 1.3% when only 65 samples were analyzed [16]. Therefore, it was presumed that the number of samples should be increased to obtain better accuracy. For the new analysis, in total 367 measurements were made and the same number of reference samples was collected. The measurements were made in 5 different lots, and the thickness and moisture properties of the biomaterial mat were varied between the lots by adjusting the settings of the belt filter press differently for each of them. 

The reference samples were collected from the mat after the corresponding resonant frequencies were recorded. The area and thickness of the samples were measured in the laboratory prior to oven-drying and they were on average 96 mm^2^ and 14.3 mm, the average volume of the samples being 138 cm^3^. The samples were oven-dried and weighed before and after in the laboratory for the gravimetric determination of the moisture content. For wood, the standard oven-drying temperature is typically 100–105 °C [17]. There is no standard for the biofuel in question but as a precaution, the temperature of the oven was set only to 80 °C so that volatilization of organic matter would not occur; the biomaterial consists of a high amount of microbes and some chemicals that are added before burning. The relative water content (wet-basis) of the reference samples determined using (1) varied between 47–67%.

## 3. Results

In an online microwave measurement, the sensor does not measure a gravimetric moisture content of discrete “samples”, but instead it represents a volumetric measurement of a moving mat. The effective volume of the measurement would be defined as the thickness of the mat multiplied by the measurement footprint, which is determined by the width of the center conductor (200 mm) of the strip line cavity resonator. The measurement footprint is slightly larger than the area of the center strip, the half-power area being approximately 300 mm × 100 mm [14]. Therefore, the average area of the reference samples (96 mm^2^) was clearly smaller than the footprint of the measurement. However, the accurate volume element of each measurement point cannot be defined because the thickness changes constantly and the sensor does not obtain information about the rate of this change.

Normally one would find a correlation between the relative water content and resonant frequency shift. However, in this current application, the thicker the material, the lower its relative water content, but the absolute water content is higher than for a thinner mat. This is related to the operation of the belt filter press, and it is necessary to model the water content as an absolute quantity such as thickness of the equivalent water layer [16]. The equivalent water layer is not tightly dependent on the thickness of the material. In principle, the same amount of dry material is inserted in the press constantly, and pressing affects the density and water content of the mat. If the belt filter press is adjusted to produce a thinner mat, this consequently results in a mat which is denser. Alternatively, a less tightly pressed mat is not as dense but contains more water.

The equivalent water layer corresponds to the amount of water per surface area in each sample, which essentially the sensor measures, considering the assumption of the negligible effect of the dry material. The height of the equivalent water layer is equivalent to the mass per area of water through the following equation:

(4) $$h_{eq}[\mathrm{mm}]=\frac{V_{water}}{A_{sample}}\underset{=}{\Delta }\frac{m_{w}[g]}{10^{3}[g/m^{3}]}\cdot \frac{1000}{A_{sample}[m^{2}]}=m_{aw}[\mathrm{kg}/m^{2}].$$

Finding a suitable correlation between the water content and $\varepsilon_{r}^{\prime }$ is a complicated task and depends on many parameters, e.g., on frequency; for pure water, the real part of permittivity is independent of frequency below the relaxation frequency.

**Figure 5:**
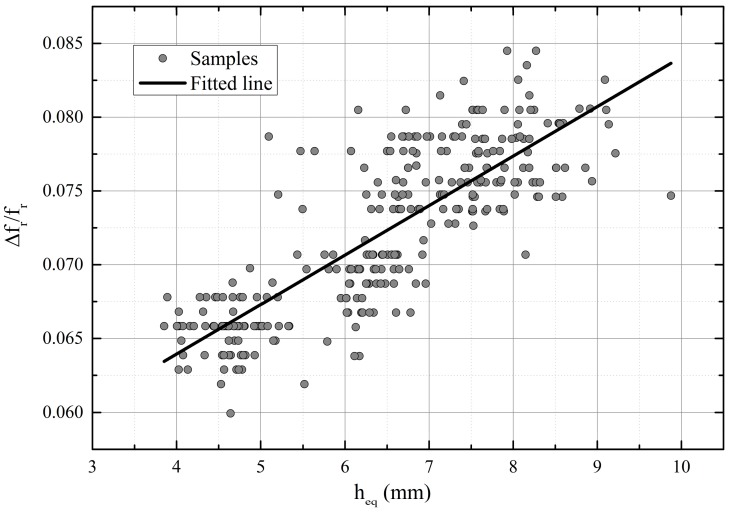
The relative resonant frequency shift vs. thickness of the equivalent water layer.

**Figure 6:**
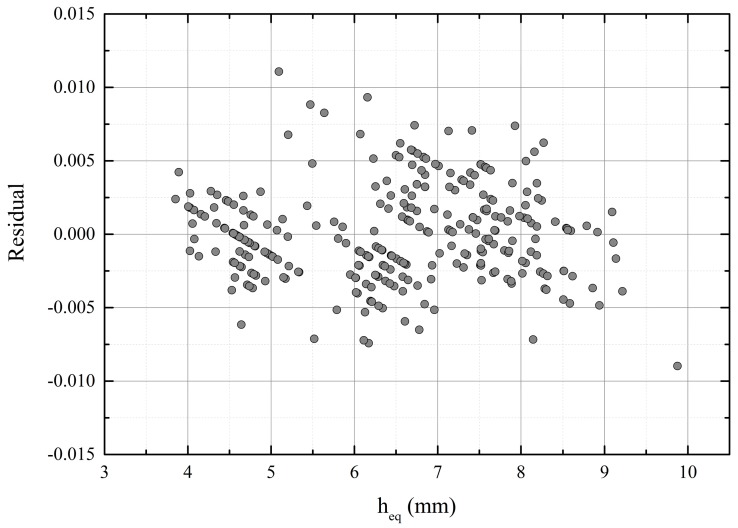
Residual plot for linear fit.

Statistical correlation was determined between the measured parameter and the material parameter in [18], where the moisture content of single soy beans was measured using a resonator. The dependency between the resonant frequency shift and water content was found to be linear for a constant dry mass. Ref. [19] reported that the relation between the moisture content and $\varepsilon_{r}^{\prime }$ (at 18 GHz) of wool was non-linear but, above 20% of moisture, the permittivity increased more steeply, following a linear trend. This kind of behavior was attributed to the bound-water effect. As a first estimate, a linear relation is assumed also between the permittivity of the biomaterial mat and the water content. In addition, the resonator sensor type is designed to support the even mode and be linear as a function of ($\varepsilon_{r}^{\prime }-1$), based on the approximation of the perturbation formula [11]. The result of the moisture measurement is presented in Figure 5, with a linear fit. Figure 6 shows the corresponding residual plot of the linear fit.

In an on-line measurement variations due to constant changes in thickness of the moving material pose a challenge. Feasibility of the single-parameter measurement is based on an estimation that the dry material has a significantly smaller effect on the resonant frequency than water. However, changes in the thickness or density also cause uncertainty in the measurement of the water content. The thicknesses of the samples were measured after collecting them from the mat, and they varied between 7 mm to 21.4 mm.

Another analysis of the results was made to diminish the uncertainty due to the large variations in the thickness of the material. The number of samples was reduced so that fewer water content levels share the same resonant frequency shift. For each measurement data set (5 in total), the average height of the water layer is different because of the settings in the filter press system. The average thickness of the mat with corresponding standard deviation (STD), and thickness of the equivalent water layer is listed for each series in Table 1. The range of resonant frequency shifts was determined so that at maximum one standard deviation was allowed from the average height of the equivalent water layer for each dataset and, consequently, the number of samples was reduced to 235. The measurement resolution of the resonant frequency was 0.35 MHz, because the number of points was 201 in the band 310 to 380 MHz. The thickness of the equivalent water layer varied now only approximately ±1 mm per 0.35 MHz or per 0.1 percentage points shift of the relative resonant frequency, which was essentially the “resolution” in Figure 5.

**Figure 7:**
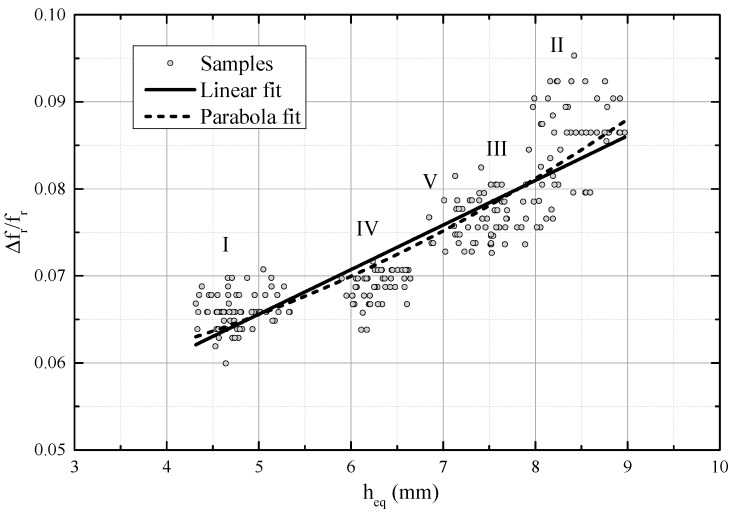
Thickness of the equivalent water layer vs. the relative resonant frequency shift with a reduced number of samples. A linear fit (R^2^ = 0.78) and a parabola fit (R^2^ = 0.82).

**Figure 8:**
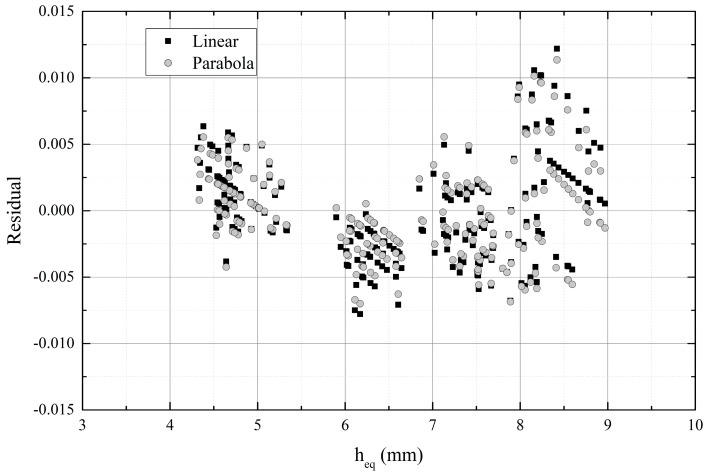
Residuals for parabola and linear fit.

Figure 7 presents the thickness of the equivalent water layer across the relative resonant frequency shift. Both a linear fit and a parabola fit were determined and they gave R^2^ = 0.78, R^2^ = 0.82, respectively. Figure 8 shows the residual of the linear and parabola fit and the corresponding parameters are listed in Table 2. Data sets I and IV are distinguishable but sets II, III and V lie partly on top of each other between water levels 7 mm and 9 mm. Between the water levels 8 mm and 9 mm, the resonant frequency shift is higher than would be expected based on the linear fit or parabola fit. Such a phenomenon was observed in [20], where wet paper was measured with a similar strip line resonator, even though the dependency was expected to be linear based on theory. Their assumption was that at higher values of $\varepsilon_{r}^{\prime }$, determination of the moisture content, and thickness is more uncertain of very wet paper (>50%).

Table 2 lists the parameters for linear fit of original data (Figure 5), as well as the linear and parabola fit after reducing the number of data points according to Table 1.

## 4. Conclusions

### 4.1. Discussion

This paper carried out new measurements and calculations as part of a feasibility study on the moisture measurement of biofuel. The employed sensor type is an existing design which is in commercial use for moisture measurement of veneer. The research was conducted in collaboration with a Finnish paper factory. When the biomaterial is highly inhomogeneous in particular, point-like resonant frequency measurements are not informative about the water content of the material as such, but instead statistical processing and analysis of the data is required.

The operation of the belt filter press is not directly related to actual measured resonant frequency shifts. As a compromise for taking into account thickness variation of the samples and uneven distribution of the relative moisture content, the water content in each sample was modelled as an equivalent water layer.

The operation of the press is not designed so that the thickness could be adjusted to remain constant and, therefore, addition of density and thickness sensors would be imperative to make the measurement more reliable. In terms of other environmental factors, the moisture measurement is made in a factory environment, where the humidity does not vary greatly. The moisture sensor is intended for applications within the range 40–60% water content. Small ambient humidity changes would be calibrated with the measurement of an empty resonator and remain below noise level i.e., not affect the resonant frequency more than the measurement resolution.

The number of reference samples was increased to 367 during this measurement campaign. Comparing the standard error with *n* = 65 ([16]) and *n* = 367, ($STD/\sqrt{n}$) has reduced from 0.02% to 0.003%. Nevertheless, variation of water layers was high, more than ±1 mm for the same resonant frequencies. Then, at a maximum one standard deviation was allowed from the average height of the equivalent water layer for each dataset and the number of samples was further reduced to 235. Both a linear fit and a parabola fit were determined and they gave R^2^ = 0.78 and R^2^ = 0.82, respectively.

### 4.2. Future Work

There are a number of improvements to the protocol that could be suggested, if this was to be used in the future. In general, the assumption of an equivalent water layer is too simplified, because the thickness and impact of permittivity of the dry material is ignored completely. In reality, the thickness of the material changes randomly as the mat passes the sensor.

Another simplification in the presented measurement configuration is that the resonant frequency shift was measured only once per measurement footprint. The mat was moving constantly through the sensor so it was not possible to take, for example, three recordings at the same location. This would be possible employing only one sensor, if the belt was stopped during the measurement. In general, measurements of reference samples of a very inhomogeneous material in a laboratory do not correlate perfectly with factory tests, even though the performance of the strip line cavity sensor was verified earlier in [12,14,20].

Repeatability of one point-like measurement is poor, as the material changes constantly. Even though the mat has supposedly similar moisture and thickness conditions along the lateral direction, deploying a sensor array of similar sensors that measure and record the resonant frequency shift simultaneously could improve accuracy. Impurities in the material cause a widening of the resonance curve and difficulty determining the exact resonant frequency. Operation of the belt filter press does not allow for a constant thickness of the mat. In conclusion, it would not appear sufficient to assume that the variation in thickness of the biofuel mat has a negligible influence when using a single parameter moisture measurement.

## Figures and Tables

**Table 1:** Average thickness of the equivalent water layer with corresponding standard deviation (STD) and the average thickness of the mat for the measurement series I–V.

Measurement Series	I	II	III	IV	V
Average thickness of equivalent water layer (mm)	4.79	8.40	7.74	6.31	6.91
STD (mm)	0.49	0.59	0.63	0.39	0.64
Average thickness of the mat (mm)	9.2	20.5	16.0	10.5	15.4

**Table 2:** Parameters of the linear and parabola fit.

Equation	Linear (Orig. Data Figure 5)	Linear (Datasets I–V, Figure 7)	Parabola (Datasets I–V, Figure 7)
	*y* = A + B*x*	*y* = A + B*x*	*y* = A + B*x* + C*x*^2^
A	0.0505	0.03998	0.05554
B	0.0034	0.00512	0
C	-	-	4.0073 × 10^−4^
R^2^	0.67	0.78	0.82
Residual min	−0.00898	−0.0078	−0.007
Residual max	0.01108	0.01218	0.01136
